# Long-term, basin-scale salinity impacts from desalination in the Arabian/Persian Gulf

**DOI:** 10.1038/s41598-022-25167-5

**Published:** 2022-11-29

**Authors:** Francesco Paparella, Daniele D’Agostino, John A. Burt

**Affiliations:** 1 Division of Sciences, New York University Abu Dhabi, Abu Dhabi, United Arab Emirates; 2 Arabian Center for Climate and Environmental Sciences, New York University Abu Dhabi, Abu Dhabi, United Arab Emirates; 3 Water Research Center, New York University Abu Dhabi, Abu Dhabi, United Arab Emirates

**Keywords:** Climate sciences, Climate-change impacts, Environmental sciences, Environmental impact, Ocean sciences, Physical oceanography, Biooceanography

## Abstract

The nations on the shoreline of the Arabian/Persian Gulf are the world’s largest users of desalination technologies, which are essential to meet their freshwater needs. Desalinated freshwater production is projected to rapidly increase in future decades. Thus, concerns have been raised that desalination activities may result in non-negligible long-term, basin-wide increases of salinity, which would have widespread detrimental effects on the Gulf marine ecosystems, with ripple effects on fisheries, as well as impacting the desalination activities themselves. We find that current yearly desalinated freshwater production amounts to about 2% of the net yearly evaporation from the Gulf. Projections to 2050 bring this value to 8%, leading to the possibility that, later in the second half of the century, desalinated freshwater production may exceed 10% of net evaporation, an amount which is comparable to interannual fluctuations in net evaporation. With the help of a model we examine several climatological scenarios, and we find that, under IPCC’s SSP5-8.5 worst-case scenarios, end-of-century increases in air temperature may result in salinity increases comparable or larger to those produced by desalination activities. The same scenario suggests a reduced evaporation and an increased precipitation, which would have a mitigating effect. Finally we find that, owing to a strong overturning circulation, high-salinity waters are quickly flushed through the Strait of Hormuz. Thus, even in the worst-case scenarios, basin-scale salinity increases are unlikely to exceed 1 psu, and, under less extreme hypothesis, will likely remain well below 0.5 psu, levels that have negligible environmental implications at the basin-wide scale.

## Introduction

The shoreline of the Arabian/Persian Gulf (hereafter ‘Gulf’) is shared between eight nations, many of which have experienced a rapid economic development in the last 30 years, accompanied by dramatic population growth and urbanization. Abundant availability of freshwater resources is a necessary condition to sustain any development. Within the Gulf, only Iran and Iraq can rely on naturally occurring watercourses for their freshwater needs. Therefore, it is not surprising that the countries on the southern Gulf shore have been among the earliest adopters of desalination technologies, which today supply most of the freshwater used in the Gulf region^1^.

**Figure 1:**
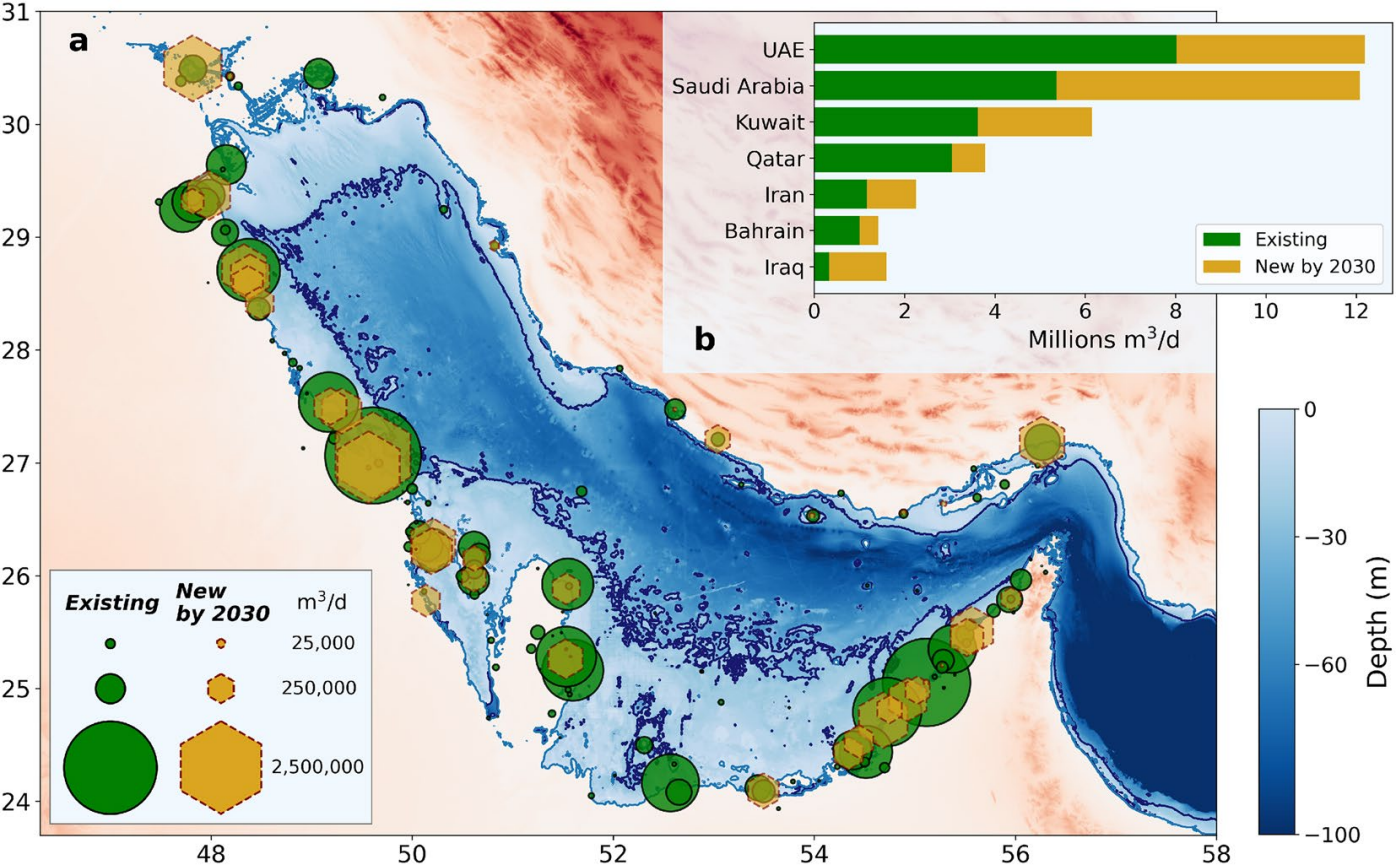
(**a**) Bathymetry of the Gulf region according to the GEBCO 2021 dataset. Elevation zero is marked in light blue, the − 30 m isobath in dark blue. Existing and future (in construction, approved and planned by 2030) desalination plants impinging on the Gulf are marked, respectively by circles and hexagon markers. The marker area is proportional to the plant desalination capacity. Plants closer than ~10 km are represented as a single plant with the combined capacity. (**b**) Existing and future desalination capacity per country. Oman has been omitted because its desalination capacity in the Gulf amounts to only 5150 m$^{3}$ day$^{-1}$, with no plans for expansion. Data from^2^. Map produced with Python 3.10.6: https://www.python.org/.

As of today, 45% of global freshwater desalination production is concentrated within the Gulf, and the region is home to the biggest desalination plant complexes in the world^3^. Figure 1 shows the position and the associated freshwater production capacity of all the desalination plants that are currently (March 2022) in operation (i.e., ‘existing plants’) and of those that are expected to be in operation by 2030 (‘future plants’, including plants in construction, approved or planned).

Although desalination is fundamental to support life and wellness within the Gulf^4–7^, many concerns have been raised in relation to its environmental impacts^8–13^. Local impacts include the impingement and entrainment of marine organisms at the water intake and the discharge of heated, hypersaline, chemically polluted and hypoxic brines (the main by-product of desalination) at the outfalls, which can have direct negative effects on the marine flora and fauna. The local-scale action of brines in increasing temperature (for flash and multi-effect distillation plants) and salinity (for all plants) is somewhat clear^3,12,14^. On the other hand, the extension of this phenomenon at the basin-wide scale, and its synergistic effect with warming and increases in salinity due to climate change, would depend on the characteristics of the circulation of water masses in the basin on whose shores the plants occur.

The Gulf is a shallow marginal sea (mean depth ~30 m) where evaporation exceeds precipitation and river run-off^15^. Connected to the Indian Ocean through the narrow Strait of Hormuz (‘Hormuz’), the Gulf is characterized by a reverse-estuarine circulation, where dense, saline waters (39–40 psu) outflow through the deeper part of Hormuz, and lighter, fresher Indian Ocean Surface Waters (‘IOSW’, 36–37 psu) inflow at shallower levels^16–18^. The exchange flux is relatively weak in comparison to that of other similar semi-landlocked basins (e.g., Red Sea, Mediterranean Sea) and averages to about 0.15 Sv^19^. The Gulf already experiences extreme environmental conditions for a subtropical sea, with temperatures reaching 36 $^{\circ }$C in summer and typical salinities of 42 psu in the southern shallows^15,20^. Therefore, the possibility that desalination activities may result in non-negligible, basin-wide salinity increases, with potentially negative environmental and economic impacts, should be carefully addressed.

Indeed, the current high salinity of the Gulf has already been linked to reduced biodiversity of corals and echinoderms^21–24^ and reduced size at maturity in fish^25^. Size reduction is associated with the higher cost of osmoregulation at higher salinity, which reduces the energy available for growth, reproduction and maintenance^26^. Crucially, decreasing fish sizes will impact fisheries’ productivity due to reduced fish fecundity and biomass production^27^. Furthermore, feed water salinity is one of the major determinants of the operating costs of a desalination plant, therefore a substantial increase in salinity would also negatively impact the economic viability of freshwater production through desalination^28,29^.

Recently, several modelling studies have attempted an estimation of the increase of salinity at basin-wide spatial scales due to desalination within the Gulf^13,30–36^. However, these studies only consider a very limited number of scenarios, mostly focusing on quantifying the excess salinity due to present-day desalination volumes. They do not consider the possible future climate change effects, nor the likely increases in the desalination volumes, with the notable exception of^13^ which extrapolates both the desalination volumes and the climatic conditions to mid-21st century. Due to their heterogeneity, these studies are not easily comparable, and their results differ significantly from each other. For present-day desalination capacity, all studies report salinity increases less than 0.5 psu, with the exception of the analytical model^36^ which predicts Gulf-wide salinity above 50 psu, and of the general circulation model^13^ which reports widespread salinity increases in excess of 1 psu in the offshore regions, and of 3 psu or more in shallow coastal areas. None of these works identify specific physical mechanisms that favor or hamper the salinity buildup. Some studies report slight changes in the circulation of the Gulf^33,35^, however, due to the limited interval of time spanned by their simulations, they could not disentangle the relative contribution of the desalination plant discharge and that of the naturally-occurring interannual variability of salinity.

**Figure 2:**
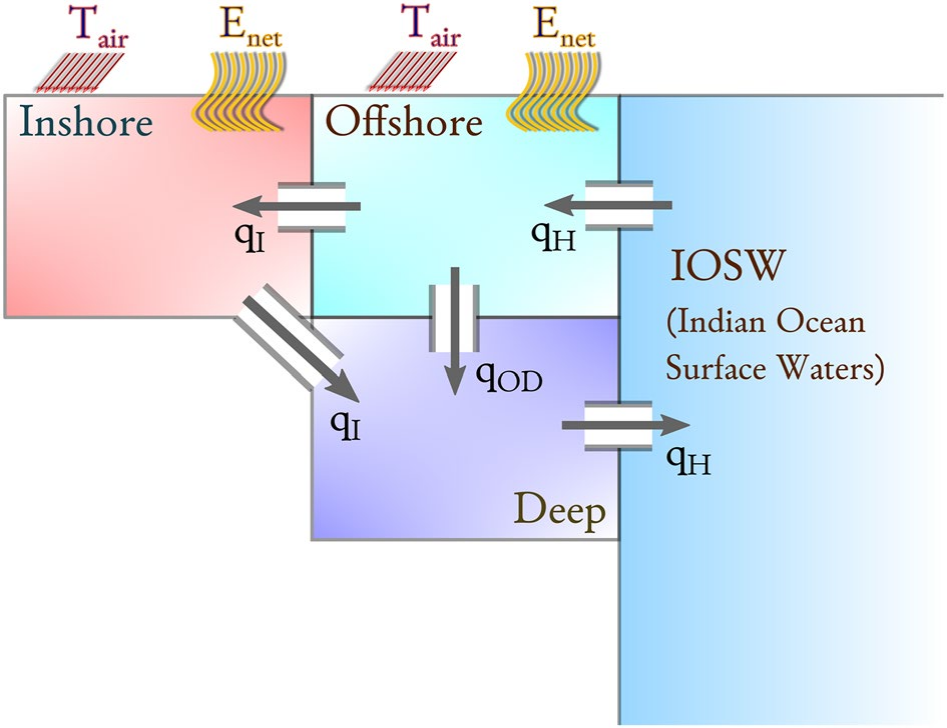
Conceptual scheme of the box model (see “Methods” for the model equations). Volume fluxes ($q_{I}$, $q_{H}$, $q_{OD}$) between the Indian Ocean, inshore, offshore, and deep boxes are driven by the density differences between the water masses in adjacent boxes. The inshore and offshore boxes are subject to evaporation and heat exchanges with the atmosphere. The flux $q_{H}$ quantifies the water exchange between the Indian Ocean and the Gulf through the Strait of Hormuz.

More generally, the existing literature has paid little attention to identifying simple, usable benchmarks for gauging the size of the desalination fluxes, in order to achieve a qualitative picture that may guide further quantitative analysis. An estimate of the freshwater desalination production in the Gulf (e.g. Fig. 1) is of economic and sociological relevance, but its absolute value conveys little environmental information unless it is contrasted against a meaningful comparison term. A qualitative indicator that applies to any substance release in the water is the ratio of the flushing times associated with the substance release (by desalination, in our case) and with the substance removal (by transport through Hormuz). We further observe that both desalination and evaporation have similar effects (they draw freshwater away, leaving sea salt mass behind, resulting in increased sea salinity), with the only difference that the first is an anthropogenic process, which occurs exclusively onshore, while the latter is a natural process that occurs both onshore and offshore. Given that the salinity of the Gulf is primarily determined by the balance between evaporation and the inflow of fresher waters through the Strait of Hormuz^16^, net evaporation (that is, evaporation minus precipitation) as well as the magnitude of its natural interannual variability, appear to be the natural comparison terms for the size of the desalination fluxes.

Our quantitative analysis does not attempt a detailed forecast of the future. Rather, its goal is to separate the plausible from the implausible, and to shed light on the primary physical processes that favor or hamper salinity build-ups at large scales. To this end we rely on a simple model for representing the overturning circulation of the Gulf. This allows us to identify a credible range of variability for future changes of salinity in the Gulf, by exploring several scenarios. In particular, we explore the effects, individually or combined, of a climatological increment in temperature, and of a climatological increment or decrement in evaporation. In addition, we also formulate scenarios based on hypothetical mechanisms regulating the flow through the Strait of Hormuz. Within each scenario, we examine a large interval of desalinated freshwater production volumes, which includes today’s volumes, estimates for 2030, and projections for 2050. The highest volume that we consider is 120 millions m$^{3}$ day$^{-1}$, which is above any known future projection, but is technologically attainable.

Our model uses an idealized partitioning of the Gulf in three boxes (Fig. 2): an *inshore* one, representing the waters from the coastline to the − 30 m isobath; an *offshore* one, for the waters beyond the − 30 m isobath from the surface to − 30 m of depth; a *deep* one, for the offshore waters below − 30 m of depth (Fig. 1 shows the − 30 m isobath^37^). The model treats the boxes as homogeneous and interconnected. The *inshore* box also accounts, in an aggregate way, for the contribution of hypersaline coastal areas such as the Gulf of Salawah and the Abu Dhabi lagoons, without, however, explicitly resolving them. The *deep* and *offshore* boxes are in communication with a reservoir representing the Indian Ocean Surface Waters. Volume fluxes are driven by density differences of the seawater in adjacent boxes. Heat and evaporation fluxes are prescribed at the surface. This modeling technique follows the seminal work by Stommel on the Atlantic overturning circulation^38^ and it has been established as an excellent approach for exploratory studies of thermohaline overturning circulation, also when including atmospheric feed-backs^39–42^. The box model approach allows to treat the brine input of desalination plants as a withdraw of freshwater, akin to evaporation. The only parameter relevant for the model is thus the total volume of desalinated water produced in the unit of time. This makes the model results independent of the particular desalination technology being used and, in particular, of the specific brine / freshwater ratio of each plant. See “Methods”, below, for further details and for the model equations.

## Results

### Climatology of evaporation fluxes

**Figure 3:**
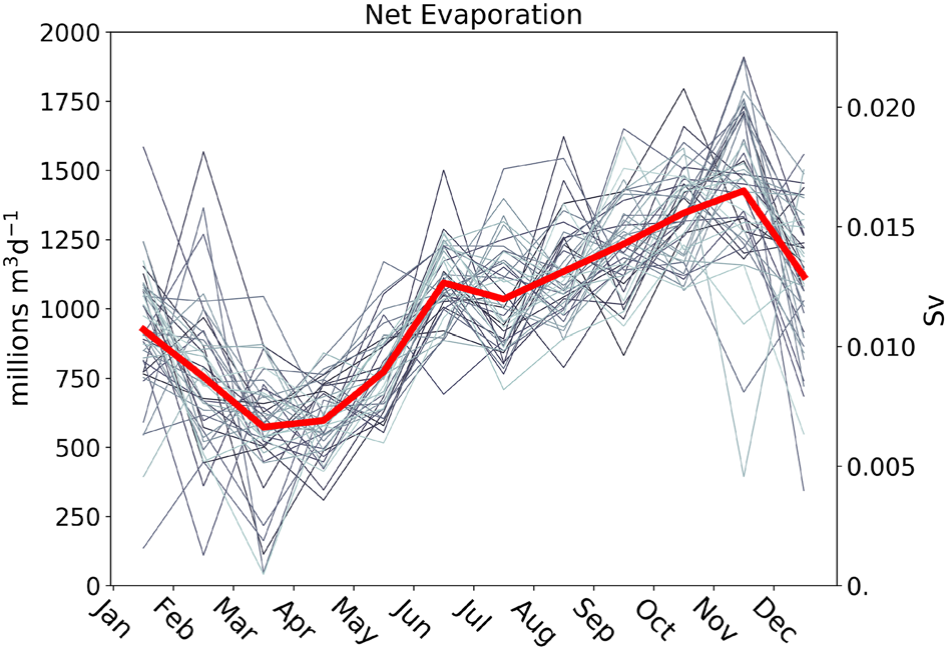
Monthly averaged net evaporation (evaporation minus precipitation) fluxes over the Gulf. The thick red line is the average over the time interval 1979–2021. The thin gray lines refer to the individual years (darker color corresponds to older year). The left axis reports the flux in millions of cubic meters per day, the right axis reports the same flux in Sverdrup (1 Sv = one million cubic meters per second). Data from the ERA5 reanalysis.

Evaporation from the Gulf waters reaches a minimum in March–April, and slowly climbs to a maximum in November^43,44^. The monthly averaged net evaporative flux over the Gulf in the years 1979–2021 is shown in Fig. 3 (data from the ERA5 reanalysis^45^). On average, at the annual minimum the net evaporation is slightly above 500 million m$^{3}$ day$^{-1}$, and at the annual maximum it almost attains 1500 million m$^{3}$ day$^{-1}$. The daily average net evaporation over the time interval 1979–2021 is 1000 million m$^{3}$ day$^{-1}$. This amount is compatible with the estimates obtained from the measurement of the volume fluxes through the Strait of Hormuz ($1105\pm 270$ million m$^{3}$ day$^{-1}$^19^, data from December 1996 to March 1998). The seasonal cycle of net evaporation shows a substantial interannual variability (Fig. 3). Fluctuations of several hundreds of millions of m$^{3}$ day$^{-1}$ above or below the monthly average value are often observed, in particular in autumn and winter. The phase of the cycle is only partially determined by the winter precipitation (which, on average, reaches its maximum in December and January, with a flux of 250 million m$^{3}$ day$^{-1}$), and is primarily affected by winds and by the temperature difference between the sea and the air, with the sea being generally cooler than the air in late winter, and warmer than the air (thus more subject to evaporation) in autumn^15,16^.

**Figure 4:**
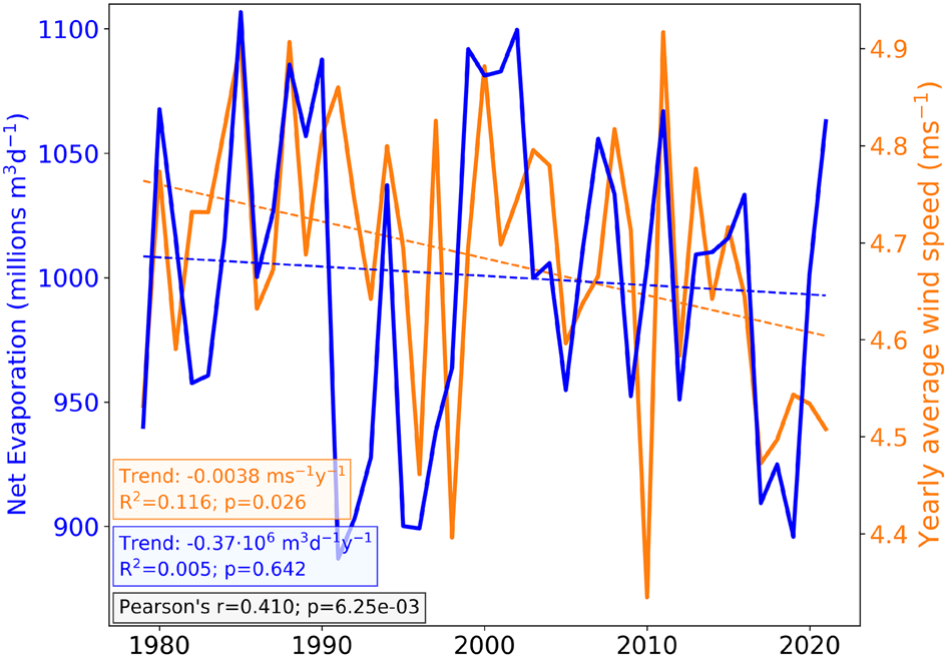
Yearly averaged net evaporation (blue solid line, left axis) and yearly averaged wind speed (orange solid line, right axis) over the Gulf. The dashed lines show the least-square linear fit to the data. The insets report the trend (slope of the linear fit) and the associated p-value respect to the null-hypothesis of zero trend. The Pearson’s correlation coefficient of the evaporation and wind time series and its p-value respect to the null-hypothesis of no correlation are also reported. Data from the ERA5 reanalysis^45^.

Figure 4 shows the Gulf’s annual mean net evaporation and wind speed. The net evaporation shows interannual fluctuations of ±10% around its mean value. The wind shows a weak, but significant, downward trend ($p<0.05$); the net evaporation trend is not statistically significant. The two quantities are significantly correlated ($r=0.41,\,p<0.01$) confirming that changes in wind speed strongly affect evaporation in the Gulf^46,47^. (Omitting precipitation, the correlation of wind and evaporation alone increases to $r=0.57,\,p=0.0001$; evaporation alone also does not show a significant trend).

### Comparison with the desalination fluxes

The current estimate of the desalinated water production in the Gulf is 22.6 million m$^{3}$ day$^{-1}$^2^ which amounts to 2.3% of the average daily net evaporation flux in the period 1979–2021. Including all of the known future plants (Fig. 1) the desalination capacity grows to 39.5 million m$^{3}$ day$^{-1}$, that is, 4% of the average net evaporation flux. This should be a fairly accurate assessment of the desalination fluxes by circa 2030. It is projected that by 2050 the desalination capacity may reach 80 million m$^{3}$ day$^{-1}$^13^. Therefore, the possibility that during the second half of the 21st century desalination fluxes may reach, and possibly surpass, 10% of the evaporation fluxes should be considered as a plausible hypothesis.

**Table 1:** End-of-century projected changes of present-day atmospheric conditions over the Gulf by the IPCC AR6 CMIP6 models.

	Wind speed	Precipitation	Air temperature
SSP1-2.6	− 2%	+ 5%(*)	+ 1.3 C
SSP5-8.5	− 5%	+ 25%(*)	+ 4.7 C

Two of the five IPCC scenarios reported here: SSP1-2.6 (sustainability) and SSP5-8.5 (fossil-fueled development) correspond, respectively, to a pervasive, world-wide curbing of GHG emission, and to continuing world-wide economic growth driven by an ever-increasing consumption of fossil fuels. (*) Individual CMIP6 models show a substantial and conflicting variability in precipitation change estimates.

It is useful to note that the surface area inshore of the − 30 m isobath is 58% of the total area of the Gulf. Therefore, currently desalination draws from the inshore region a volume of fresh water equal to roughly 4% of that drawn by net evaporation and this amount will grow to about 7% including future capacity (up to 2030). Afterwards, during the second half of the 21st century the freshwater draw may rise above 15% of the inshore evaporation fluxes. In summary, today’s desalination fluxes are dwarfed by the monthly fluctuations of net evaporation (Fig. 3) but may become comparable with the present-day interannual fluctuations of net evaporation (Fig. 4) in the second half of the 21st century. The scenarios discussed in the IPCC sixth assessment report (AR6)^48^ show a continuing downward trend in wind speed (Table 1), which in the extreme scenario SSP5-8.5 matches that observed in Fig. 4, and is less rapid in the sustainability scenario SSP1-2.6. Declining winds may lead to declining evaporation, and the projected (albeit highly uncertain) increase in precipitation may further decrease net evaporation. While these effects would counterbalance the desalination fluxes, the box model (below) reveals that the largest climate-change impact on salinity may come from the projected increase in air temperature.

The water volume inshore of the − 30 m isobath is about 1900 billion m$^{3}$, and amounts to 24% of the total volume of the Gulf^37^. At current capacity, the total freshwater uptake from desalination in one year is about 0.4% of the inshore volume, and if the uptake increased to 120 million m$^{3}$ day$^{-1}$, it would exceed 2% of it. Desalination is thus associated with a timescale that ranges from 250 years under present conditions to less than 50 years if desalination reaches the highest end of freshwater production considered here (120 million m$^{3}$ day$^{-1}$, which exceeds current projections for 2050^13^). This time scale should be compared with the typical time scales of water exchange through the Strait of Hormuz. The flushing time is defined as the ratio between the volume of a body of water over the magnitude of its exchange fluxes. The Gulf has a volume of 7900 billions m$^{3}$, and it exchanges approximately 0.15 Sv with the Indian Ocean through Hormuz^19^ (1 Sv = 1 million m$^{3}$s$^{-1}$). This leads to an estimated gulf-wide flushing time of about 1.7 years. A related quantity is the residence time, defined as the average time necessary for a water parcel starting at a prescribed initial location to leave the body of water. We are not aware of any direct measurement of residence times (e.g. through floats) in the Gulf, thus we refer to modeling results^49^. In the shallows facing the UAE coastline, the residence time is estimated to be shorter than two years. This time increases to three years only for water parcels starting in Kuwait bay. Even the water in the Gulf of Bahrain is estimated to reach Hormuz in less than 2.5 years. For water parcels starting offshore of the − 30 m isobath, the residence time is estimated to range between three months and one year, except for starting points in the immediate proximity of Hormuz which may have residence times shorter than a month.

These qualitative results suggest that the impact of desalination fluxes on the Gulf-wide salinity balance may be undetectable under current conditions, but may become measurable in some future scenarios.

**Figure 5:**
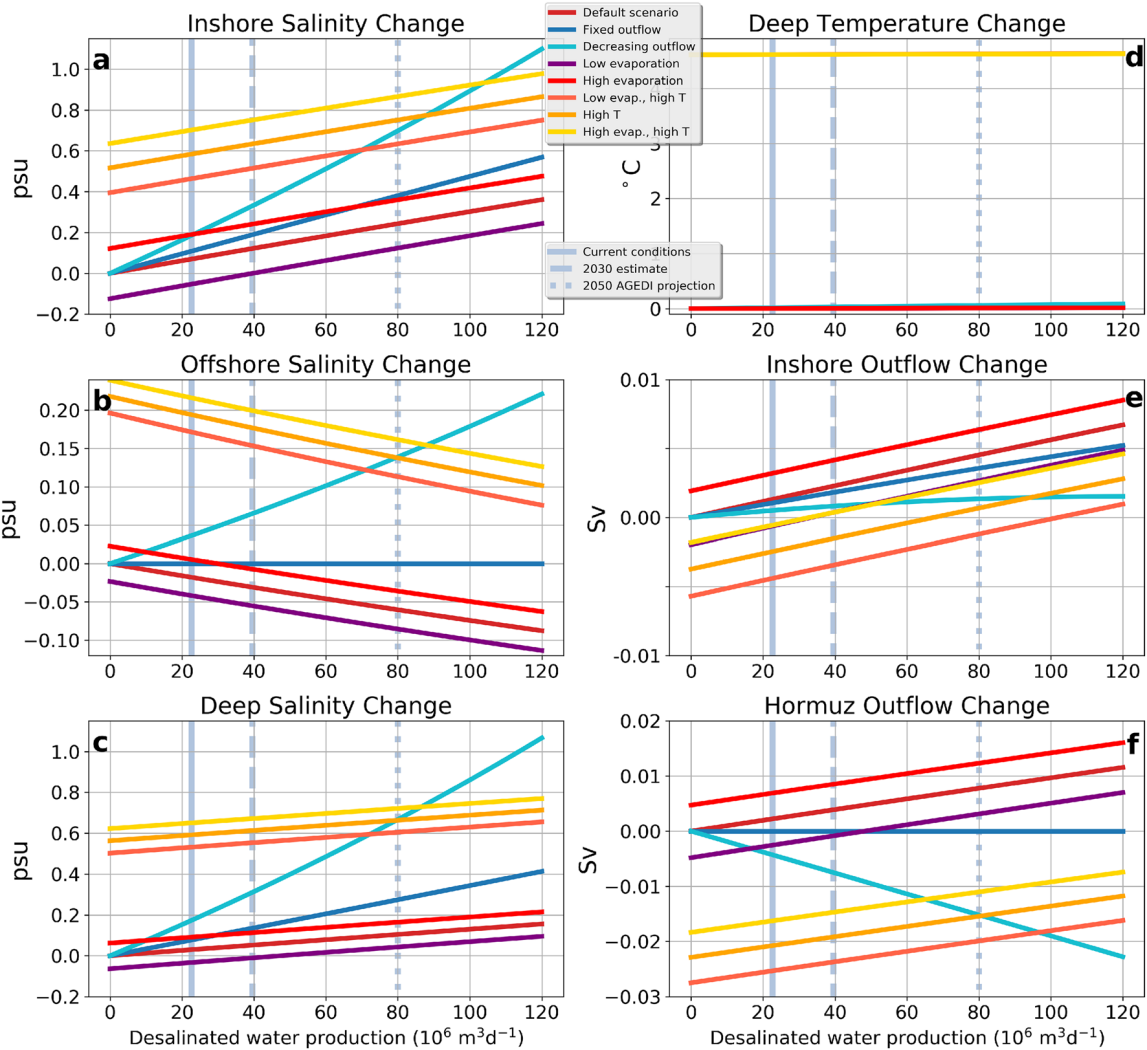
Yearly-averaged changes with respect to the baseline run (present-day climate with no desalination) as a function of the desalination fluxes. The panels show the change in salinity in the inshore (**a**), offshore (**b**) and deep (**c**) boxes. Temperature changes in the deep box are shown in panel (**d**). Panels (**e**) and (**f**) show, respectively, changes in the outflow from the inshore to the deep box and through Hormuz. The vertical gray lines mark the present-day desalination fluxes (continuous), the expected desalination fluxes by 2030 (dashed) and AGEDI’s projected desalination fluxes in 2050 (dotted).

### Desalination scenarios

A baseline model run aims at reproducing present-day conditions with no desalination (see “Methods” for details, parameters and validation). Eight scenarios have been developed, based on hypotheses involving the dynamics of the Hormuz outflow and climate-change related phenomena. The change in key quantities with respect to the baseline run is then quantified as a function of the desalination fluxes (Fig. 5).

The “**default**” scenario assumes a constant, present-day climate. Salinity in the inshore and deep boxes increases, respectively, by 0.4 psu and 0.2 psu when desalination fluxes reach 120 m$^{3}$ day$^{-1}$. This is accompanied by a *decrease* in salinity in the offshore box. This is explained by the 7% increase of the outflow $q_{H}$ through Hormuz (out of the deep box, see Fig. 2), which at the surface is matched by an inflow of an equal volume of fresher water from the Indian Ocean into the offshore box.

It has been suggested that the Hormuz outflow may be, at least partially, hydraulically controlled^19^, that is, be in a state where the flow rate is not determined by downstream density gradients alone but also by the topography of the Strait^50^. Although hydraulic control for the Gulf has not been confirmed, it is interesting to formulate a “**fixed outflow**” scenario, where the flow out of Hormuz is held constant, and thus is not affected by any salinity or density changes due to desalination activities. In this scenario, the salinity in the inshore box increases 1.6 times more than in the default scenario, and, in the deep box, salinity increases twice more than in the default scenario. Because the inflow from the Indian Ocean is fixed, the salinity of the offshore box does not change.

A recent regional ocean circulation modeling effort, in a future climate scenario for the Gulf, reports of a slightly decreased deep outflow associated with an increase in the deep water density in the central part of the Gulf^51^. The study does not attempt to identify a plausible mechanism causing this unexpected inverse relationship between density and volume flows. Nevertheless, we decided to formulate a “**decreasing outflow**” scenario where the Hormuz outflow is prescribed to decrease with increasing desalination fluxes, up to a 15% reduction with respect to present-day values for desalination fluxes of 120 m$^{3}$ day$^{-1}$. Not surprisingly, this scenario produces the fastest increase of the salinity in the inshore and deep boxes, exceeding 1 psu salinity increase with respect to the baseline run at the highest end of the desalination range. This is also the only scenario in which the salinity of the offshore box increases.

The following two scenarios, “**low evaporation**” and “**high evaporation**” use the baseline parameters, but the yearly averaged net evaporation is, respectively, decreased and increased by 5%. A 5% decrease in net evaporation would correspond to extrapolating the trend of Fig. 4 to the end of the century, and is coherent with IPCC’s SSP5-8.5 CMIP6 projections (Table 1). In both cases, the model shows a response to desalination analogous to that of the default scenario, but offset in the direction of the mitigation of the desalination effects in the case of low evaporation, and in the opposite direction for high evaporation.

The IPCC projections without substantial emission reductions also suggest strong atmospheric heating over the Gulf. Thus, we have a “**high temperature**” scenario that uses the baseline parameters, except for the yearly average air temperature, which is incremented by 5 $^{\circ }$C. High air temperatures are propagated from the inshore and offshore boxes into the deep one, which warms by more than 4 $^{\circ }$C. This, in turn, reduces the density difference between the deeper Gulf waters and the IOSW, causing a reduction of the outflow through the strait. Salinity may then build-up in the inshore and deep box more easily than in the default scenario, reaching a 0.9 psu increase with respect to the baseline run.

The high temperature scenario is further modified in the “**low evaporation, high temperature**” and “**high evaporation, high temperature**” scenarios, by accompanying the temperature increase with a 5% decrease and increase (respectively) of the yearly average net evaporation. The outcomes of these two scenarios straddle the high temperature results. As before, a decrease in net evaporation mitigates the effects of desalination, and an increase intensifies them.

In the baseline run, and in all of the scenario runs, the volume fluxes go from the offshore to the inshore box, and from the inshore to the deep box, as well as from the Indian Ocean reservoir to the offshore box, and from this into the deep box and back to the Indian Ocean reservoir. That is, the density differences between the boxes always push the flow in the direction of the arrows in Fig. 2, even though this is by no means imposed by the model, which would allow fluxes in the opposite directions, if the density differences so dictated.

## Discussion and conclusions

Desalination is an irreplaceable source of freshwater for many countries around the Arabian / Persian Gulf. In this region, desalination has scaled up to levels not attained elsewhere. Today, the freshwater production from plants drawing from Gulf waters exceeds 2% of the freshwater removed from the Gulf by net evaporation fluxes. In the second half of this century, this amount may increase beyond 10% of net evaporation. These are staggering levels that elicit legitimate questions on the sustainability of desalination activities.

In this paper we focus on the possibility of salinity build-ups in the Gulf, and especially its shallower regions (inshore of the − 30 m isobath). We do not discuss what may happen in the short term and in the vicinity of a large desalination plant, a subject that has already drawn extensive attention in the literature, but we examine long-term, basin-wide effects with eight scenarios that combine a wide range of desalination fluxes with specific hypothesis on the future evolution of the regional climate and the dynamical nature of the overturning circulation in the Gulf. To this end, we eschewed the use of full-fledged general circulation models. Previous studies, reviewed in the introduction, obtained contrasting results, and made little progress in elucidating the mechanistic chains of causes and effects that produced the reported increases in salinity (or lack thereof). Here, we use a box model specifically developed to represent the Gulf’s overturning circulation. This allows us to identify the boundaries of what should be taken as possible and realistic, and identify the external conditions and internal chains of events that produce a given result.

Our main finding is that the salinity of the Gulf is crucially linked to the deep overflow through the Strait of Hormuz. This may not be a surprising conclusion, given the geography of the Gulf, but we are not aware of other studies clearly stating this link and exploring its consequences. The salinity increase inshore of the − 30 m isobath directly due to desalination is estimated to be no more than 0.4 psu, even at the highest end of the desalination freshwater production that we considered, and which far exceeds the projections for 2050. With present-day desalination fluxes the increase is less than 0.1psu, an amount that is practically undetectable. Desalination-driven salinity increases above 1 psu may be attained only under the hypothesis that the Hormuz outflow decreases with increasing salinity gradient across Hormuz. The authors have no knowledge of any evidence (theoretical or observational) that could support the presence of such an inverse relationship in the Gulf. The limited increase in salinity predicted by the model is coherent with the fact that the flushing time of the Gulf (the main time scale of response to evaporation or desalination forcings) is just 1.7 years. Thus, under present conditions, the Gulf responds to forcings on a time scale comparable with that of the interannual variability in net evaporation. Therefore, it is not surprising that a steady forcing due to desalination, with an intensity comparable to the interannual fluctuations of evaporation (about 10% of the total net evaporation) produces a salinity increase comparable with the observed interannual salinity fluctuations^18^. Furthermore, steady increases in salinity (e.g. by desalination) lead to increases of the flow through the Strait of Hormuz, which shortens the flushing time. This negative feedback further limits the effect of desalination on salinity.

A second important factor is air warming due to climate change. The vigorous, density-driven overturning circulation of the Gulf rapidly takes ventilated coastal waters to the deepest part of the basin, where they are expelled from the Gulf through the Strait of Hormuz^16,52^. The model shows that even the extreme heating occurring in the SSP5-8.5 scenario is not enough to offset the formation of dense water in the Gulf’s shallower regions, which, even in that scenario, sinks into the deepest part of the Gulf. This process rapidly transmits to the deep Gulf waters any regional atmospheric heating trend, leading to a situation where the Gulf deep waters are substantially lighter than in present-day conditions. This reduces the density gradient across Hormuz and, therefore, the volume of the deep outflow. Atmospheric heating hence facilitates a salinity build-up. However, even when accompanied by a 5% increase in net evaporation, in the model this build-up is contained within 1 psu, even for the highest levels of desalination. Some care is necessary in interpreting these scenario results, because in the box model the description of the stratification in the deeper part of the Gulf is extremely rough, being approximated by two homogeneous water masses of fixed volume (the “deep” and the “offshore” boxes), stacked one on top of the other. The quantitative details of our findings shall be verified with a realistic ocean circulation model. However, both our model and the available observations^16^ suggest that the overturning circulation is driven by processes occurring in shallow coastal areas (the “inshore” box), where the hypothesis of vertical homogeneity appears to be reasonable. We are thus confident that more realistic models, even in a climate change scenario, would qualitatively show the same dynamics as the present model.

We must stress that the above discussion is based on the SSP5-8.5 climate scenario from IPCC, which is highly pessimistic and unlikely to reflect what will actually happen^53^. Therefore our climate-change scenarios identify upper bounds, rather than most likely values for future salinity increases in the Gulf. Furthermore, in our scenarios the IOSW are assumed not to warm, which exaggerates the reduction of the density gradient through Hormuz. Finally, for such an extreme scenario IPCC projects a decrease in wind speed over the Gulf, and, possibly, an increase in precipitation (Table 1), which would, in turn, translate into a reduction of net evaporation, which would somewhat mitigate the effect of heat on salinity, as in the scenario “low evaporation, high temperature”.

A possible source of uncertainty in our estimates is due to the so-called *produced water*. This is saline water pumped out of petroleum and gas wells together with the hydrocarbons. It is separated in-situ and then either re-injected into the well or purified and released at sea. We are not aware of any published data on produced water in the Gulf, nor of its salinity. However, it is estimated^54^ that the yearly global produced water flux into the oceans is about 700 million m$^3$. This corresponds to a global discharge about 2 million m$^3$/day, equivalent to a single very large desalination plant. We thus assume that the impact of produced water from oil wells to the salinity of the Gulf is a minor contribution with respect to the total impact of desalination plants, although more data is necessary to stand this hypothesis on firm ground.

The Gulf’s waters are already characterized by high seasonal and interannual variability in salinity^15,18^, therefore the expected 1 psu salinity increase predicted by our worst-case scenario (i.e., ‘decreasing outflow’) is unlikely to have a significant impact on the Gulf’s marine life. Indeed, flora and fauna currently inhabiting the Gulf represent a subset of the western Indian Ocean biota^55^ and are already adapted to withstand extreme temperature and salinity and large intra- and inter-annual fluctuations^56,57^. Obviously, our statement that, even in an extreme climate change scenario the gulf-wide salinity will not raise to alarming levels, should not be construed as a claim that those scenarios would not have an impact on the biogeochemistry and ecology of the Gulf. For example, already in present day-conditions there is mounting evidence for the occurrence of hypoxia, seasonally in the deepest part of the Gulf^58^ and occasionally in shallow coastal reefs^59^. The warming of the deepest part of the Gulf suggested by the model also raises concerns. Furthermore, impacts of increased salinity associated with brine discharges have been reported elsewhere for corals^60^, seagrasses^61–63^ and fishes^64^, while empirical data from Gulf species are still nearly entirely lacking, although hypersalinity has been associated with reduced diversity of corals and echinoderms (seastars, urchins and relatives) in the Gulf^21–24^. Salinity, indeed, may play a significant role in structuring the Gulf’s biodiversity, and some contrasting effects have already been reported across different organisms. In fishes, for example, salinity increases and salinity variation were identified as one of the potential causes determining dwarfism in Gulf populations, suggesting potential increases in osmoregulatory costs and associated reduced energy availability for growth which will eventually lead to reduced population fecundity, replenishment and long-term persistence^25^. On the other hand, in stony corals and sea anemones, Gulf’s high salinity has been linked to increased thermotolerance and decreased bleaching susceptibility, suggesting a potentially important link between osmoadaptation to high salinities and tolerance to thermal stress^65–67^. Thus, further studies are urgently needed to fully clarify how the mutual interplay of high salinity, high temperatures and their large fluctuations, drives or otherwise affects the unique physiological adaptations that shape the various Gulf marine ecosystems, both under current conditions and under future modeling scenarios.

In summary, we found that for salinity inshore of the − 30 isobath to substantially increase above 1 psu by the end of this century it would require the action of physical processes as-yet unknown. On the contrary, unless the air temperature increased as much as in the SSP5-8.5 IPCC scenario, it is reasonable to expect salinity increases to be contained within 0.5 psu, a value comparable with seasonal and year-to-year fluctuations. This contained salinity increase is unlikely to directly affect marine life, however it may play a contributing role in an intertwined network of physical drivers and ecological responses whose understanding is necessary to properly identify outstanding vulnerabilities and propose meaningful mitigation strategies.

## Methods

**Table 2:** Baseline model parameters.

Symbol	Definition	Value	Ref/comments
$T^{\dagger }$	IOSW temperature	20 $^{\circ }$C	^16–18^
$S^{\dagger }$	IOSW salinity	36.5 psu	^16–18^
$V_{I}$	Volume of inshore box	$1.9\cdot 10^{12}$ m$^{3}$	^37^
$V_{O}$	Volume of offshore box	$3.3\cdot 10^{12}$ m$^{3}$	^37^
$V_{D}$	Volume of deep box	$2.7\cdot 10^{12}$ m$^{3}$	^37^
*f*	Gulf surface area fraction in offshore box	0.42	^37^
$k_{I}$	Inshore flux constant	40 Sv	^39^; regulates inshore salinity
$k_{H}$	Hormuz flux constant	95 Sv	^39^; regulates flux through Hormuz
$\tau _{O}$	Atmospheric relaxation time, offsh.	30 days	^39^; regulates temperature difference between inshore and offshore
$\tau _{I}$	Atmospheric relaxation time, insh.	$\tau _{O}{\displaystyle \frac{V_{I}}{V_{O}}}$days	^39^; regulates temperature difference between inshore and offshore
$\alpha$	Thermal expansion coefficient	$1.5\cdot 10^{-4}$ $^{\circ }$C$^{-1}$	^39^
$\beta$	Haline contraction coefficient	$8\cdot 10^{-4}$ psu$^{-1}$	^39^
*D*	Desalination flux	0 m$^{3}$ day$^{-1}$	For each scenario, this is varied in the range $0-120\cdot 10^{6}$m$^{3}$ day$^{-1}$.
$\overline{E_{net}}$	Average net evaporation	$1000\cdot 10^{6}$ m$^{3}$ day$^{-1}$	^45^; see Fig. 3
$A_{net}$	Net evaporation yearly amplitude	$\pm 0.45\overline{E_{net}}$ m$^{3}$ day$^{-1}$	^45^; see Fig. 3
$\overline{T^{*}}$	Average air temperature	26.5 $^{\circ }$C	^45^
$A^{*}$	Air temperature yearly amplitude	$\pm 0.32\overline{T^{*}}$ $^{\circ }$C	^45^
$\rho _{ref}$	Reference density	1000 kg m$^{-3}$	

Our box model partitions the Gulf waters into three idealized, homogeneous boxes (Fig. 2): *inshore*, representing the waters between the shoreline and the − 30 m isobath, *offshore*, representing the waters offshore of the − 30 m isobath from the surface down to − 30 m of depth, and *deep*, representing the offshore waters below − 30 m of depth. Two dynamically distinct regions exist in the Gulf: a deeper one dominated from spring to autumn by vigorous mesoscale vortices, and a shallow one dominated by tides and density-driven currents^15,18,49,68,69^. The − 30 m isobath appears to be a reasonable separating threshold between the two regions^18,68^ and thus we chose it to define the boxes. Density differences drive volume fluxes between the three boxes. The offshore and deep boxes are also in contact with the Indian Ocean Surface Waters (IOSW), which, for simplicity, are modeled as an infinite water reservoir at constant temperature and salinity. The temperature of the offshore and inshore boxes relaxes to the air temperature, according to a seasonal cycle^70^. Evaporation and precipitation fluxes are prescribed to match mean, amplitude and phase of the seasonal cycle shown in Fig. 3. In the inshore box, desalination fluxes are also prescribed, and are used as a control parameter. The model equations are:

$$\begin{aligned} {\dot{T}}_{I}&= \frac{T^{*}-T_{I}}{\tau _{I}}+\frac{H(q_{I})q_{I}(T_{O}-T_{I})}{V_{I}}-\frac{\left( 1-H(q_{I})\right) q_{I}(T_{D}-T_{I})}{V_{I}}\\ {\dot{T}}_{O}&= \frac{T^{*}-T_{O}}{\tau _{O}}+\frac{H(q_{H})q_{H}(T^{\dagger }-T_{O})}{V_{O}}-\frac{\left( 1-H(q_{OD})\right) q_{OD}(T_{D}-T_{O})}{V_{O}}-\frac{\left( 1-H(q_{I})\right) q_{I}(T_{I}-T_{O})}{V_{O}}\\ {\dot{T}}_{D}&= \frac{H(q_{I})q_{I}(T_{I}-T_{D})}{V_{D}}+\frac{H(q_{OD})q_{OD}(T_{O}-T_{D})}{V_{D}}-\frac{\left( 1-H(q_{H})\right) q_{H}\left( T^{\dagger }-T_{D}\right) }{V_{D}}\\ {\dot{S}}_{I}&= S_{I}\frac{(1-f)E_{net}+D}{V_{I}}+\frac{H(q_{I})q_{I}(S_{O}-S_{I})}{V_{I}}-\frac{\left( 1-H(q_{I})\right) q_{I}(S_{D}-S_{I})}{V_{I}}\\ {\dot{S}}_{O}&= S_{O}\frac{fE_{net}}{V_{O}}+\frac{H(q_{H})q_{H}(S^{\dagger }-S_{O})}{V_{O}}-\frac{\left( 1-H(q_{OD})\right) q_{OD}(S_{D}-S_{O})}{V_{O}}-\frac{\left( 1-H(q_{I})\right) q_{I}(S_{I}-S_{O})}{V_{O}}\\ {\dot{S}}_{D}&= \frac{H(q_{I})q_{I}(S_{I}-S_{D})}{V_{D}}+\frac{H(q_{OD})q_{OD}(S_{O}-S_{D})}{V_{D}}-\frac{\left( 1-H(q_{H})\right) q_{H}\left( S^{\dagger }-S_{D}\right) }{V_{D}} \end{aligned}$$

where $T_{I}$, $T_{O}$, $T_{D}$, $S_{I}$, $S_{O}$, $S_{D}$ are functions of time and represent, respectively, the temperatures and salinities of the inshore, offshore and deep boxes. *H* is the Heaviside step function (whose value is one for positive arguments and zero otherwise). The volume fluxes $q_{I}$, $q_{OD}$, $q_{H}$ are defined as

$$\begin{aligned} q_{I}&= k_{I}(\rho _{I}-\rho _{D})\\ q_{H}&= k_{H}(\rho _{D}-\rho _{IOSW})\\ q_{OD}&= q_{H}-q_{I} \end{aligned}$$

They are taken as positive in the direction of the arrows in Fig. 2. We use a linear equation of state for the densities $\rho _{i}=\rho _{ref}(1-\alpha T_{i}+\beta T_{i})$ for $i\in \{I,D,IOSW\}$. We are supported in our choices of the expressions for $q_{I}$ and $q_{H}$ by observational evidence that volume fluxes in the Gulf are driven by bottom density differences^16^. The expression for $q_{OD}$ is then dictated by volume conservation. In two of the eight scenarios discussed above, the flux $q_{H}$ through the Strait of Hormuz is externally imposed, and does not depend on the density difference across the Strait. In the “fixed outflow” scenario the flux is held constant: $q_{H}=0.152$ Sv. In the “decreasing outflow” scenario the flux decreases linearly with the desalination fluxes according to $q_{H}=0.152(1-1.25\cdot 10^{-3}D)$. The net evaporation $E_{net}$ and the reference atmospheric temperature $T^{*}$ are functions of time defined as:

$$\begin{aligned} E_{net}(t)=&\overline{E_{net}}+A_{net}\sin \left( \frac{2\pi }{Y}(t-\phi _{net})\right) \\ T^{*}(t)=&\overline{T^{*}}+A^{*}\sin \left( \frac{2\pi }{Y}(t-\phi ^{*})\right) \end{aligned}$$

where *Y* is the length of a year, $\phi _{net}$ is chosen to have maximum evaporation in mid-October, $\phi ^{*}$ is chosen to have maximum air temperature in mid-July. The other constants that appear in all of the above equations are defined in Table 2, together with their value and supporting references. A baseline model run with the parameter values of Table 2, after an initial transient, produces yearly averaged salinities of 41.8 psu, 37.7 psu, 39.5 psu, for, respectively, the inshore, offshore and deep boxes. Both the inshore and the deep box show yearly fluctuations of 0.8 psu. The salinity in the offshore box fluctuates by 0.4 psu. Density in the inshore box ranges from 1028.2 kg m$^{-1}$ (reached in August) to 1030.7 kg m$^{-1}$(reached in February); density in the offshore box ranges from 1025.1 kg m$^{-1}$(August) to 1027.4 kg m$^{-1}$(February); density in the deep box ranges from 1027.3 kg m$^{-1}$(October) to 1028.3 kg m$^{-1}$(April). The simulated flow $q_{H}$ through the Strait of Hormuz has a yearly average of 0.152 Sv, with seasonal fluctuations of $\pm 0.048$ Sv. Despite the simplified nature of the model, and of its crude representation of the vertical stratification, these values are coherent with the observed values^15,16^ (in particular, see Figs. 7a–d, 8 in Swift & Bower). The model yields a flow $q_{I}$ from the inshore to the deep box ranging from 0.025 Sv (July) to 0.11 Sv (January), which is in very good agreement with the OGCM simulations of Al-Shehhi et al.^52^ (see their Fig. 10). Each scenario, except for the “default” one, modifies some of the parameters of Table 2 as specified in the “results” section. For each scenario, the desalinization flux is varied in the range $0-120\cdot 10^{6}$ m$^{3}$ day$^{-1}$. At each desalination level the simulation is run until a stationary annual cycle is obtained. The change in yearly average from the baseline run is shown in Fig. 5.

## Data Availability

The box model software is publicly available at https://doi.org/10.5281/zenodo.6519835. The ERA5 reanalysis data are publicly available from the Copernicus data repository at https://cds.climate.copernicus.eu/cdsapp#/dataset/reanalysis-era5-single-levels?tab=overview. The desalination capacity data shown in Fig. 1 are property of DesalData https://www.desaldata.com. Restrictions apply to the availability of these data, which were used under license for the current study, and so are not publicly available. Data are however available from the authors upon reasonable request and with permission of DesalData.
